# RETRACTION: The Effect of the Use of Postoperative Antibiotic Prophylaxis Compared with Non‐Use for Stented Distal Hypospadias Repair Wound: A Meta‐Analysis

**DOI:** 10.1111/iwj.70217

**Published:** 2025-02-11

## Abstract

**RETRACTION**: TangR. , WanL. , YiZ. , LuoY. , WeiX. , WangS. , and XiaoC. , “The Effect of the Use of Postoperative Antibiotic Prophylaxis Compared with Non‐Use for Stented Distal Hypospadias Repair Wound: A Meta‐Analysis,” International Wound Journal 20, no. 8 (2023): 3073–3080, 10.1111/iwj.14182.37095731 PMC10502256

The above article, published online on 24 April 2023, in Wiley Online Library (http://onlinelibrary.wiley.com/), has been retracted by agreement between the journal Editor in Chief, Professor Keith Harding; and John Wiley & Sons Ltd. Following an investigation by the publisher, all parties have concluded that this article was accepted solely on the basis of a compromised peer review process. In addition, the investigation found unattributed textual overlap between this article and another article by different authors [1]. The editors have therefore decided to retract the article. The authors did not respond to our notice regarding the retraction.

**Reference:**

[1] ChuaM. E. , KimJ. K. , RiveraK. C. , MingJ. M. , FloresF. , and FarhatW. A. , “The Use of Postoperative Prophylactic Antibiotics in Stented Distal Hypospadias Repair: A Systematic Review and Meta‐Analysis,” Journal of Pediatric Urology 15, no. 2 (2019): 138–148, 10.1016/j.ejogrb.2019.11.026.30527683

**RETRACTION**: R. Tang , L. Wan , Z. Yi , Y. Luo , X. Wei , S. Wang , and C. Xiao , “The Effect of the Use of Postoperative Antibiotic Prophylaxis Compared with Non‐Use for Stented Distal Hypospadias Repair Wound: A Meta‐Analysis,” International Wound Journal 20, no. 8 (2023): 3073–3080, 10.1111/iwj.14182.37095731 PMC10502256

The above article, published online on 24 April 2023, in Wiley Online Library (http://onlinelibrary.wiley.com/), has been retracted by agreement between the journal Editor in Chief, Professor Keith Harding; and John Wiley & Sons Ltd. Following an investigation by the publisher, all parties have concluded that this article was accepted solely on the basis of a compromised peer review process. In addition, the investigation found unattributed textual overlap between this article and another article by different authors [1]. The editors have therefore decided to retract the article. The authors did not respond to our notice regarding the retraction.

## Reference

[1] M. E. Chua , J. K. Kim , K. C. Rivera , J. M. Ming , F. Flores , and W. A. Farhat , “The Use of Postoperative Prophylactic Antibiotics in Stented Distal Hypospadias Repair: A Systematic Review and Meta‐Analysis,” Journal of Pediatric Urology 15, no. 2 (2019): 138–148, 10.1016/j.ejogrb.2019.11.026.30527683

